# Burnout syndrome in healthcare workers during the COVID-19 pandemic: a systematic review

**DOI:** 10.47626/1679-4435-2022-849

**Published:** 2022-03-30

**Authors:** Vinicius S. T. Meira-Silva, Anna Cecilia T. N. Freire, Danielle P. Zinezzi, Fernanda C. R. Ribeiro, Georgia D. Coutinho, Isabela M. B. Lima, Isabella C. Crispi, Juliana D. Porto, Laís G. P. Silva, Luiz Henrique A. Miranda, Maria Giullia F. Zurita, Victor Hugo R. Belerique, Yasmin T. Bandoli

**Affiliations:** 1 Universidade Estácio de Sá - Centro I Presidente Vargas, Rio de Janeiro, RJ, Brazil.; 2 Centro de Saúde Escola Lapa, Universidade Estácio de Sá, Rio de Janeiro, RJ, Brazil

**Keywords:** professional burnout, occupational health, healthcare personnel, COVID-19, pandemics

## Abstract

There is evidence that harm to the mental health of healthcare workers has occurred during the pandemic caused by COVID-19. The burnout syndrome is a form of exhaustion that occurs in occupational settings and is a condition caused by long-term stress in the workplace. The objectives of this systematic review of observational studies were to present data from research into the prevalence of burnout syndrome in healthcare workers during the COVID-19 pandemic and observe its prevalence among frontline workers. The search was conducted on the MEDLINE, LILACS, and Embase databases from 2019 to May of 2021 and returned 538 publications, which underwent a two-stage process of selection by independent peers, resulting in selection of a sample of 29 articles. Data were then extracted and synthesized for presentation in narrative form. Cross-sectional designs were more prevalent (n = 26) than longitudinal studies (n = 3). The sample included research from 19 different countries, with one Brazilian study. A wide range of different instruments were administered by study authors to assess burnout syndrome, the most common of which was the Maslach Burnout Inventory (n = 13). The prevalence of burnout syndrome in the studies varied from 76 to 14.7%. Data on the relationship between development of burnout syndrome and working on the frontline were controversial. The lack of standardization of burnout syndrome assessment was a source of considerable difficulty, compromising comparability of the results, and should therefore be targeted for improvement by researchers. We suggest that more investigations should be conducted into prevalence and the associated factors of risk and protection.

## Introduction

A new coronavirus, the severe acute respiratory syndrome coronavirus 2 (SARS-CoV-2), emerged towards the end of 2019 in Wuhan, capital of the Chinese province of Hubei. The virus spread rapidly, causing a global public health crisis, which was declared a pandemic by the World Health Organization (WHO) in March of 2020. A little more than 1 year later, more than 3.5 million deaths had been recorded worldwide, with more than 450 thousand in Brazil alone.^1^

In addition to the physical risks of the disease caused by SARS-CoV-2, harm to mental health has been observed in a large proportion of the population, even in uninfected people. Fear of infection, compounded by the rapid spread and lack of knowledge about the disease can make members of the public more susceptible to psychological suffering.^2^

Studies indicate that anxiety and stress levels increased significantly among healthy people and that preexisting symptoms in people with mental health disorders have been exacerbated. One phenomenon observed in the context of uncertainty about the virus was panic and anxiety crises, amplified by fear of contamination of oneself and of others and fear of death.^3^

The mixture of workplace stress factors and personal fears linked to COVID-19 pandemic appears to have placed an enormous psychological burden on healthcare teams. One of the most important disorders observed in workers is burnout syndrome (BS), a form of exhaustion that occurs in occupational settings and is a condition caused by long-term stress in the workplace.

Burnout syndrome was first described by Herbert Freudenberger in 1974. The social psychologist Christina Maslach developed the classic definition of BS, characterizing it in three dimensions: emotional exhaustion (feelings of overload and emotional and physical exhaustion), depersonalization (feelings of indifference or distance in relation to others in the workplace), and low personal accomplishment (feelings of incompetence or lack of achievement and productivity at work).^4^ Schaufeli & Greenglass^5^ summarized BS as “a state of physical, emotional and mental exhaustion that results from long-term involvement in work situations that are emotionally demanding.”

The WHO only recognized BS as a chronic disease in 2019, and there is a clear lack of research and publications on the subject prior to this date.^6^

Risk factors for development of BS may be situational or individual. Different aspects of the working environment can be cited as situational factors, for example, workload, availability of support, and a person’s degree of control over their work.^7^ The individual aspects are related to a person’s primary personality structure, such as idealism, perfectionism, timidity, insecurity, and difficulty coping with stressful situations, and to their prior experience, (weak) support network, etc.^8^

There are a number of other characteristics linked in the literature with development of BS in healthcare workers in the context of the pandemic, such as coping difficulties, providing care in high-risk units, and contact with infected patients. In counterpoint, resilience, trust in protective measures, organizational support, and training have been identified as protective factors.^9^

The consequences of this disorder can be harmful to both professionals and patients. There is a possibility of increased frequency of medical errors, negligence, team member turnover, suicide, and abuse of alcohol and drugs by physicians, in addition to patient dissatisfaction, and lower quality of care.^10-12^ Higher rates of employee absenteeism are also related to BS, which has also been linked to development of cardiovascular diseases, musculoskeletal pain, and depressive symptoms etc.^13^

There is much yet to be learned about which interventions are most effective for treating BS, but individual and group activities have been studied, including mindfulness and group therapy, in addition to the effect of reducing working hours.^14,15^ On the other hand, the importance of investing in prevention and worker protection is indisputable.^16^

In other recent health crises with significant impact, such as the Middle East respiratory syndrome (MERS) and the severe acute respiratory syndrome (SARS) epidemics, a prevalence of BS was observed equating to around one third of healthcare workers.^17^

## Objectives

The objective of this systematic review of observational studies was to present data from Brazilian and international research into the prevalence of burnout syndrome in healthcare workers during the COVID-19 pandemic and observe its possible association with working on the frontline.

## Methods

### Study design

This study adopts the systematic review of observational studies model. The research question was defined using the PECO method (an acronym formed from P: Population/patient; E: Exposure; C: Comparison/control; and O: Outcome), providing the foundation for the database searches.

### Study identification and selection

The bibliographic search was conducted in May of 2021 on the following databases: MEDLINE, LILACS, and Embase. The search strategy employed combinations of the main descriptors related to mental health, healthcare workers, and COVID-19, including publications from 2019 up to the search date.

A two-stage study selection process was conducted independently by peers. The elements these researchers analyzed in the first stage were limited to reading title and abstract and identifying at least one exclusion criterion, at which point the publication was eliminated (Table 1). The second stage of selection required the researchers to certify that all preselected inclusion criteria were present by reading the full texts of each of the publications not eliminated in the first stage. Any disagreements between the examiners during the selection process were decided by a third analyst with more experience.

**Table 1:** Exclusion and inclusion criteria for selection of articles

Inclusion criteria	Exclusion criteria
I. Observational studies (cohort, case-control, cross-sectional, comparative, non-randomized).	I. Studies not conducted with human beings.
II. Studies of populations of healthcare workers exposed to the COVID-19 pandemic while working.	II. Absence from the study of any terms at least minimally related to the subject of the review.
III. Information on emergence of psychological disorders or symptoms during the period.	III. Literature reviews; meta-analyses; opinion pieces; editorials; letters; case reports and studies; information bulletins; chapters; guidelines; technical standards and similar; intervention studies; randomized clinical trials; studies using exclusively qualitative methodology; studies without data to characterize the target population; studies conducted by medical/health sciences students.
IV. Application of a validated or original instrument to assess burnout syndrome.	IV. Articles in languages other than English, Portuguese, or Spanish.

### Burnout syndrome assessment instruments

A number of instruments are used in the literature to assess BS. The oldest of these instruments, the Maslach Burnout Inventory (MBI), was developed in 1996 for use with humans and health services workers - for which reason it was given the subtitle Human Services Survey (MBI-HSS). Later, two other versions were developed with minor alterations for use with educators and the general public. The MBI assesses three dimensions of BS: emotional exhaustion, depersonalization, and low personal accomplishment. The scale comprises 22 items with Likert scale responses and the total score can classify the respondent at different levels of burnout. However, there is no consensus on adoption of cutoff points, which vary in the literature.^18^

The Copenhagen Burnout Inventory (CBI) is a three-part questionnaire: personal burnout, work-related burnout, and client-related burnout. These three subdivisions were adopted to enable the questionnaire to be used in many different domains. The questions related to part 1, personal burnout, are formulated in a generic manner so that anyone can answer them. This part comprises six items about a prolonged state of physical and psychological exhaustion. Work-related burnout is measured with seven items on the questionnaire and covers a prolonged state of physical and psychological exhaustion that the respondent perceives as related to their type of paid employment. Finally, client-related burnout, covered by six items, covers a prolonged state of physical and psychological exhaustion perceived at the workplace of respondents who serve clients (including patients, students, and others).^19^

There is also a diverse range of other instruments for assessment of burnout in use in the literature, including Cuestionario para la Evaluación del Síndrome de Quemarse por el Trabajo (CESQT), Mini Z Burnout Survey (Mini Z), 15 item Occupational Fatigue Exhaustion Recovery (OFERS-15), Oldenburg Burnout Inventory (OLBI), Professional Quality of Life (ProQOL), Physician Work Life Study (PWLS), and Work-related Stress Test (WRST). Self-report of presence of burnout is also used.

### Extraction and synthesis of data

The data extraction process was performed by the same researchers, noting in a spreadsheet the following data from the articles selected: (1) authorship; (2) year of publication; (3) study location; (4) description of the study population; (5) study design; (6) sample size; (7) data collection period; (8) instrument used to assess BS; (9) prevalence of burnout in the sample; and (10) prevalence of burnout in frontline workers and those doing other jobs.

A synthesis of the results obtained is presented in narrative form, in addition to analysis of descriptive statistics for certain data.

## Results and discussion

**Figure 1:**
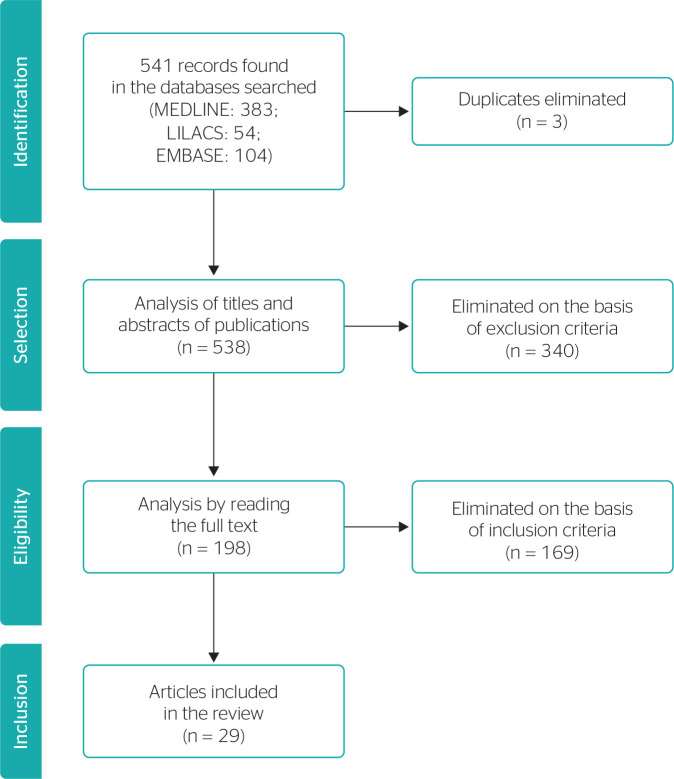
Flow diagram illustrating the phases of the systematic review.

The search of MEDLINE identified 383 references; the search of LILACS found 54 references; and 104 articles were found on Embase. Duplicate texts were removed, resulting in 538 citations (Figure 1). A total of 340 articles were eliminated in the first stage of selection and 169 were removed in the second stage, leaving a final sample of 29 publications for review.

**Figure 2:**
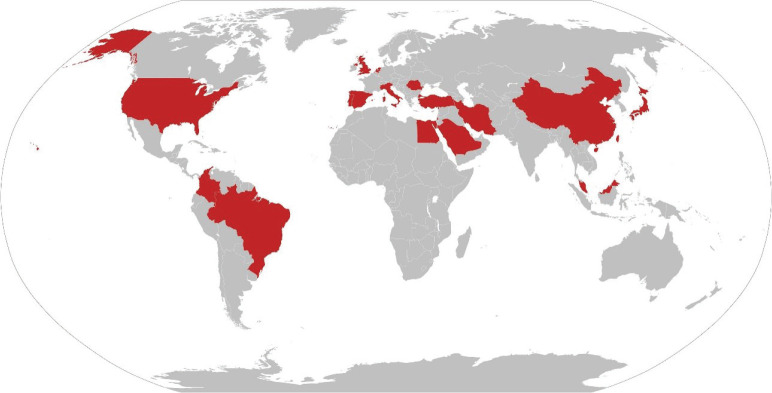
Map illustrating the studies selected for the systematic review.

Cross-sectional study (n = 26) was the most common type of study design. The sample also included longitudinal studies (n = 3). Publications from 19 different countries were selected (Figure 2), including Spain (n = 4), the United States (n = 4), China (n = 3), Italy (n = 2), and Singapore (n = 2). The populations studied were healthcare workers in a range of categories. Data for the studies were collected from February to November of 2020.

The studies reviewed used a range of different instruments to assess BS, nine of which were preexisting assessment models, while two were self report measures. The instrument most often used was the MBI (Table 2), administered in 13 studies (44.8%). However, even among studies adopting the same tool, there were differences in how authors interpreted it. Some articles defined burnout as present on the basis of high scores in one of the three dimensions assessed by the MBI (emotional exhaustion, depersonalization, or low personal accomplishment), but not all used cutoff points, presenting results in levels: low, moderate, or high.

**Table 2:** Instruments for assessment of burnout adopted by the studies selected and the number of people assessed with each one.

Instrument	Studies n (%)	Study population n	%
	(n = 29)	(n = 31,129)	
MBI	13 (44.8)	19,468	62.54
CBI	3 (10.3)	1,056	3.39
PWLS	3 (10.3)	2,797	8.99
OLBI	2 (6.8)	3,320	10.67
CESQT	2 (6.8)	896	2.88
Others	6 (20.6)	3,682	11.83

CBI = Copenhagen Burnout Inventory; CESQT = Cuestionario para la Evaluación del Síndrome de Quemarse por el Trabajo; MBI = Maslach Burnout Inventory; OLBI = Oldenburg Burnout Inventory; PWLS = Physician Work Life Study.

The characteristics of the cross-sectional studies have been compiled in Table 3.

**Table 3:** Characteristics of cross-sectional studies of burnout in healthcare workers during the Covid-19 pandemic

Authors	Country	Target population	Data collection period	n	Instrument	Prevalence of BS (%)
Gonçalves et al.^20^	Portugal	Nurses	July to November, 2020	153	CBI	NR
Nishimura et al.^21^	Japan	Physicians, nurses, and clinical engineers	November, 2020	130	MBI	24.2
Hawari et al.^22^	Jordan	Physicians, nurses, technicians, and pharmacists	April to May, 2020	1,006	PWLS	32.8
Alsulimani et al.^23^	Saudi Arabia	Physicians, nurses, and assistants	June and August, 2020	646	CBI	75.0
Torrente et al.^24^	Spain	Physicians, nurses, and assistants	April to May, 2020	643	MBI	43.4
Huang et al.^25^	Singapore	Healthcare workers in general	March to July, 2020	1,638	PWLS	21.2
Gemine et al.^26^	United Kingdom	Healthcare workers in general	April, 2020	257	CBI	NR
Lasalvia et al.^27^	Italy	Healthcare workers in general	April to May, 2020	1,961	MBI	NR
Abdelhafiz et al.^1^	Egypt	Physicians	April, 2020	220	MBI	NR
Tan et al.^28^	Singapore	Physicians, nurses, and assistants	May to June, 2020	3,075	OLBI	68.2
Manzano García & Ayala Calvo^29^	Spain	Nurses	April, 2020	771	CESQT	NR
Soto-Rubio et al.^30^	Spain	Nurses	March to April, 2020	125	CESQT	NR
Chen et al.^31^	China and Taiwan	Nurses	April, 2020	12,596	MBI	NR
Yörük & Güler^32^	Turkey	Midwives and nurses	May and June, 2020	377	MBI	NR
Firew et al.^33^	United States	Healthcare workers in general	May, 2020	2,040	MBI	NR
Mohd Fauzi et al.^34^	Malaysia	Physicians	May, 2020	1,050	OFERS-15	NR
Hoseinabadi et al.^35^	Iran	Nurses	March to April, 2020	245	OLBI	NR
Ruiz-Fernández et al.^36^	Spain	Physicians and nurses	March to April, 2020	506	ProQOL	36.0
Civantos et al.^37^	Brazil	Head and neck Surgeons	May, 2020	163	Mini Z	14.7
Matsuo et al.^38^	Japan	Healthcare workers in general	April, 2020	312	MBI	31.4
Monterrosa-Castro et al.^39^	Colombia	Family physicians	March and April, 2020	531	WRST	64.4
Rodriguez et al.^40^	United States	Emergency physicians	February to April, 2020	426	Self-report	NR
Dimitriu et al.^41^	Romania	Resident Physicians	April and May, 2020	100	MBI	76.0
Wu et al.^42^	China	Oncology physicians, and nurses	March, 2020	190	MBI	NR
Demartini et al.^43^	Italy	Healthcare workers in general	March, 2020	123	MBI	NR
Zhang et al.^44^	China	Nurses	February, 2020	646	MBI	NR

CBI = Copenhagen Burnout Inventory; CESQT = Cuestionario para la Evaluación del Síndrome de Quemarse por el Trabajo; MBI = Maslach Burnout Inventory; Mini Z = Mini Z Burnout Survey; NR = not reported; OFERS-15 = 15 item Occupational Fatigue Exhaustion Recovery Scale; OLBI = Oldenburg Burnout Inventory; ProQOL = Professional Quality of Life; PWLS = Physician Work Life Study; BS = burnout syndrome; WRST = Work-Related Stress Test.

The study with the largest sample (n = 12,596) was conducted with hospital nurses in China and Taiwan and employed the MBI instrument. The sample’s mean score was considered to show a moderate degree of burnout in the dimensions “emotional exhaustion” (19.1±10.0) and “depersonalization” (5.5±4.6), but was endorsed at higher levels by the subset of professionals in the frontline of the fight against COVID-19 (p < 0.001). The mean score in the dimension “lack of personal accomplishment” was rated low (19.0±8.4) and there was no significant difference among frontline workers.^31^

Some studies reported a high prevalence of burnout in their samples. A Romanian study conducted with physicians in hospital residency^41^ found a 76% overall prevalence of burnout. In that study, residents attached to other departments had an 86% prevalence of the condition compared to 66% among those working on the frontline (p = 0.1928).

A high prevalence of burnout was also observed among healthcare personnel working in hospitals in Saudi Arabia during the COVID-19 pandemic. Applying part of the CBI questionnaire, the study found a 75% prevalence (p < 0.0001; 95% confidence interval [95%CI] = 0.71-0.78) among these workers.^23^

A study conducted in Colombia^39^ applied the Work-related Stress Test, which explores the presence of psychosomatic symptoms related to working in a range of activities to define a diagnosis of burnout. A high prevalence (64.4%) was found in the population of family physicians assessed.

The lowest prevalence of burnout found was in a Brazilian study (14.7%) administering the Mini Z questionnaire to a small sample of head and neck surgeons from all over the country.^37^

Three of the 29 studies reviewed were longitudinal. In other words, they are research studies that analyzed the variations and characteristics of BS over a period. Each of them used a different instrument to analyze burnout (PWLS, MBI, and self-report). Two of these three studies were conducted in the United States during the same period (March to April, 2020) and both included emergency physicians in the populations studied. The third of these studies was conducted in the Netherlands over a longer period (October, 2019 to June, 2020), and the population studied was intensive care specialists (Table 4).

**Table 4:** Characteristics of longitudinal studies of burnout in healthcare workers during the Covid-19 pandemic

	Kelker et al.^45^	Kok et al.^46^	Baumann et al.^47^
Country	United States	Netherlands	United States
Target population	Emergency physicians and assistants	Intensive care professionals	Emergency physicians
Data collection period	March to April, 2020	October 2019 to June 2020	March to April, 2020
n	153	130	1,006
Instrument	PWLS	MBI	Self-report
Prevalence of BS (%)	Week 1: 30.0 Week 2: 23.0 Week 3: 20.0 Week 4: 22.0	Pre-pandemic: 23.0 Post-pandemic: 36.1	NR

CBI = Copenhagen Burnout Inventory; MBI = Maslach Burnout Inventory; NR = not reported; BS = burnout syndrome.

With regard to the prevalence of BS in each of these studies, Kelker et al.^45^ reported the weekly percentages of emergency physicians and assistants who had burnout. Since Kok et al.^46^ conducted a study that had started before the pandemic, in October of 2019, they were able to identify intensive care professionals with and without burnout during pre-pandemic and post-pandemic periods. In the first study, the percentage of professionals with burnout fell over the first weeks, but the percentage in the fourth week was 2% higher than in the previous week. In the second study, burnout levels increased by 13% during the pandemic. The other study with North-American emergency physicians^47^ did not report data on the frequency of BS.

The findings of studies that analyzed the association between development of BS and working on the frontline of the fight against COVID-19 were controversial. At least nine publications found a positive association between working on the frontline and a higher prevalence of burnout, while four others found the opposite. It is important to point out that there was a great deal of variability between the methodologies adopted in each study to test the association and the results cannot simply be compared with each other.

Certain difficulties identified during the review should be noted. They are primarily related to extraction of information about the prevalence of burnout, since there is little consistency and great variability in conceptualization of the syndrome. A large range of types of instruments with different measurement criteria are used. There is not always consensus between authors on cutoff points when using the same instrument. The lack of standardization of measurement creates a degree of heterogeneity that makes comparison of results difficult.

Observational studies are also subject to biases, such as selection bias, and their results can be influenced by confounding variables. This systematic review did not adopt procedures to assess the methodological quality of the studies included and it is therefore suggested that the results described should be read critically.

## Conclusions

This systematic review presented data from recent publications on the impacts of the COVID-19 pandemic on healthcare workers, with a focus on BS. There was considerable variation in the results of the studies reviewed. This could be a consequence of the lack of uniformity between the assessment methodologies adopted by the different authors. Standardization of instruments and their interpretations could increase comparability between studies. We recommend more investigations into prevalence and associated factors of risk and protection in order to guide development of effective public policies and interventions to prevent, treat, and rehabilitate healthcare workers, whether in periods of normality or of crisis.
